# Aggravation from Jarring the Bed

**Published:** 1895-02

**Authors:** 


### HOMŒOPATHIC PHYSICIAN,

A MONTHLY JOURNAL OF

HOMŒOPATHIC MATERIA MEDICA AND CLINICAL MEDICINE.

"If our school ever gives up the strict inductive method of Hahnemann, we are lost, and deserve only to be mentioned as a caricature in the history of medicine."—CONSTANTINE HERING.

Vol. XV. FEBRUARY, 1895. No. 2.

### EDITORIAL.

AGGRAVATION FROM JARRING THE BED.—This symptom is the great characteristic of Belladonna. It was spoken of in the editorial upon Belladonna in the October number of this journal. It will be remembered that in that editorial was related a remarkable case of prostatitis where this symptom "cropped out," and on Belladonna being given the case was quickly and perfectly cured.

Similarly the editor had a case of hip-joint disease occurring in a young girl of twelve years.

Several remedies but indifferently indicated were given without much benefit. Inflammation proceeded, and suppuration was threatening, when the patient complained of the jarring of the bed and requested us to keep away from it. She referred the effects of the shock to the diseased hip. Here was an indication for Belladonna. It was given, the inflammation subsided, and without any further treatment the patient got perfectly well *without any deformity*. To insure against any error in diagnosis Dr. Malcolm Macfarlan, of Philadelphia, who has had a large experience in hip-joint cases, was called in at the beginning of the attack, and he gave the patient a most careful and thorough examination, after which he pronounced it an undoubted case of hip-joint disease. When the patient was cured Dr. Macfarlan was again consulted to verify the cure.

Within a few days the editor has had to treat a case of gall-stone colic. Several remedies as the indications for them were exhibited were given with considerable palliation, but no permanent relief. Finally the patient complained that jarring of the bed made the pains return. Belladonna was given, and was followed by quick and permanent relief. Many other cases of such maladies as headache and facial erysipelas have developed this same symptom—aggravation from the jarring of the bed—and in every such case the administration of Belladonna in a potency has been followed by prompt and permanent relief.

This symptom was first pointed out by the late Dr. Henry N. Guernsey, under whose direction the editor of this journal began the study of medicine. Having been sent, while a student, to attend a case, and having failed to give relief, Dr. Guernsey himself visited the patient and found this indication. He gave Belladonna and the suffering was at once relieved.

Dr. Guernsey collected together and verified a great number of these indications, which he called " key-notes."

To these Dr. Lippe added more, and so a very valuable armament has been furnished to the homœopathist for the conquest of disease. Every student, and indeed every practitioner, should commit every one of these "key-notes" to memory and have them at his finger-ends for immediate use.

Dr. Lippe was always fearful that the particular attention paid to these indications would lead the practitioner insensibly into the habit of prescribing upon one symptom, and thus bring about failure.

Dr. Guernsey thought that symptoms were disposed to form natural groupings; that if the " key-note" of a remedy were present the other symptoms would also be found under the same remedy. Dr. Hering did not agree to this assertion, and preferred to call them "characteristics," as being less misleading.

The reader is thus afforded a glimpse of an old controversy between these three masters, well remembered by the old men of the school, but which has long since died out.

The great indications or "key-notes" of the remedies still remain, however, and are just as serviceable as ever. The occurrence of this one indication of "aggravation from jarring of the bed" in a case of hepatic calculus which the editor has been treating during the past week has stirred up in his mind the foregoing thoughts, which are herewith brought to the notice of the readers of this journal.

